# Telomere length heterogeneity in placenta revealed with high-resolution telomere length analysis

**DOI:** 10.1016/j.placenta.2017.09.007

**Published:** 2017-11

**Authors:** I. Garcia-Martin, A.B. Janssen, R.E. Jones, J.W. Grimstead, R.J.A. Penketh, D.M. Baird, R.M. John

**Affiliations:** a Division of Biomedicine, Cardiff School of Biosciences, Cardiff University, Cardiff, Wales CF10 3AX, UK; b Division of Cancer and Genetics, School of Medicine, Cardiff University, Cardiff, Wales CF14 4XN, UK; c Department of Obstetrics and Gynaecology, University Hospital Wales, Cardiff, Wales CF14 4XW, UK

**Keywords:** STELA, Placenta, Telomeres, Heterogeneity

## Abstract

**Introduction:**

Telomeres, are composed of tandem repeat sequences located at the ends of chromosomes and are required to maintain genomic stability. Telomeres can become shorter due to cell division and specific lifestyle factors. Critically shortened telomeres are linked to cellular dysfunction, senescence and aging. A number of studies have used low resolution techniques to assess telomere length in the placenta. In this study, we applied Single Telomere Length Analysis (STELA) which provides high-resolution chromosome specific telomere length profiles to ask whether we could obtain more detailed information on the length of individual telomeres in the placenta.

**Methods:**

Term placentas (37–42 weeks) were collected from women delivering at University Hospital of Wales or Royal Gwent Hospital within 2 h of delivery. Multiple telomere-length distributions were determined using STELA. Intraplacental variation of telomere length was analysed (N = 5). Telomere length distributions were compared between labouring (N = 10) and non-labouring (N = 11) participants. Finally, telomere length was compared between female (N = 17) and male (N = 20) placenta.

**Results:**

There were no significant influences of sampling site, mode of delivery or foetal sex on the telomere-length distributions obtained. The mean telomere length was 7.7 kb ranging from 5.0 kb to 11.7 kb across all samples (N = 42) and longer compared with other human tissues at birth. STELA also revealed considerable telomere length heterogeneity within samples.

**Conclusions:**

We have shown that STELA can be used to study telomere length homeostasis in the placenta regardless of sampling site, mode of delivery and foetal sex. Moreover, as each amplicon is derived from a single telomeric molecule, from a single cell, STELA can reveal the full detail of telomere-length distributions, including telomeres within the length ranges observed in senescent cells. STELA thus provides a new tool to interrogate the relationship between telomere length and pregnancy complications linked to placental dysfunction.

## Highlights

• STELA provides high-resolution chromosome specific telomere length profiles.
• STELA revealed no affect of sampling site, mode of delivery or fetal gender on XY telomere lengths in term placenta.
• Human placenta exhibit substantial telomere length heterogeneity.

## 1 Introduction

Telomeres are present at the ends of all mammalian chromosomes [1] and are known to maintain genomic stability avoiding degradation and fusion events [2]. In humans and other vertebrates, telomeres consist of the hexameric DNA sequence TTAGGG tandemly repeated into arrays varying in length up to 25 kb dependent on the individual or tissue analysed [1] [3]. Telomeric DNA is associated with a specific multiprotein structure called ‘shelterin’, which plays a key role in the control of telomere length and end-protection [4] [5]. Telomere repeats are synthesized by telomerase, the cellular reverse transcriptase enzyme that adds telomeric repeats to the 3′ ends of each chromosome in those cell types in which it is expressed [6] [7]. In the absence of telomerase, each time a cell divides, telomeres progressively lose TTAGGG repeats ultimately reaching a length at which they trigger a Tp53 dependent G1-S cell cycle arrest referred as replicative-senescence [8]. In the absence of a functional Tp53 response, continued cell division can result in critical telomere shortening, the induction of telomere fusion events and genomic instability that can drive tumour progression [9]. Thus telomere shortening is considered to contribute to the development of cancer, several age-related diseases and premature ageing syndromes (reviewed in Ref. [10]).

The telomere length composition at birth within the foetal genome may be important for life long health [11]. Starting life with shorter telomeres may increase disease susceptibility later in life [12]. Studies in animals and humans suggest that intrauterine exposure to adverse conditions contribute to shorter telomeres at birth [13]. The placenta is exposed to the same environmental insults as the foetus and may provide a tool to assess the effect of environmental exposures on telomeres in pregnancy [14]. Critically, shortened placental telomeres may functionally contribute to low birth weight [15], [16], [17], [18], [19], [20], [21].

**Fig. 1:**
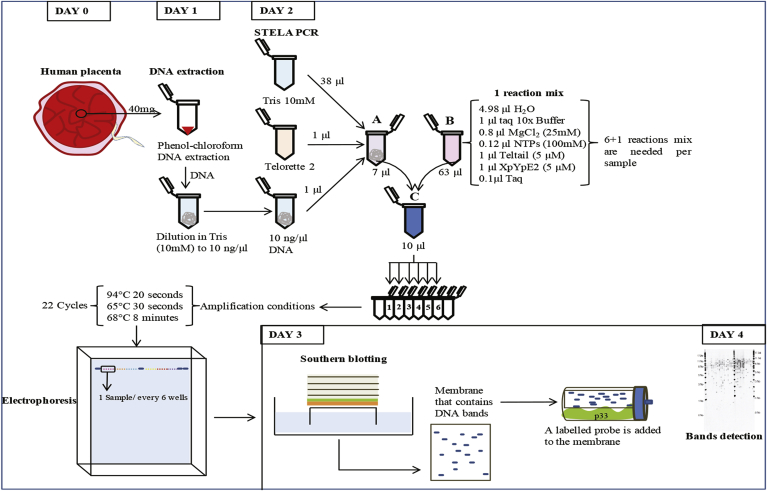
**Flow diagram of STELA protocol using placental samples**. Timeline to perform STELA technique on placental samples.

Several methods have been developed to estimate telomere length [22]. The two most widely used methods, terminal restriction fragment analysis (TRF) and quantitative polymerase chain reaction (q-PCR) are suitable for estimation of mean telomere length or telomere repeat content respectively. TRF is relatively low throughput and includes not only telomeric repeats, but also variable number of sub-telomeric sequences. Q-PCR constitutes a straightforward and suitable technique for high throughput studies, but suffers from high measurement errors [23]. High resolution telomere length measurements are obtained when used Q-FISH (Quantitative fluorescence in situ hybridization), a technique primarily used in the haematopoietic tissue that provides cell average length using metaphases [24]. Single telomere length analysis (STELA; Fig. 1), a single-molecule PCR based telomere length analysis technology that can determine the full spectrum of telomere lengths from specific chromosome ends [25]. Whilst STELA is comparatively low throughput, it is high-resolution and can detect the presence of telomeres within the length ranges that can lead to senescence, apoptosis and telomere fusion [26], [27].

STELA has the potential to provide a richer and more detailed picture of telomere length in the placenta in relation to foetal growth restriction and perhaps provide more information on the role of adverse maternal lifestyles in telomere shortening. Here we examined the patient of XpYp telomere length analysis, using STELA, with respect to placental sampling site, mode of delivery and foetal sex.

## 2 Materials and methods

### 2.1 Placental biopsies and participant selection

Study participants (N = 42) were recruited prior to delivery at University Hospital Wales and Royal Gwent Hospital as described previously [28]. Written informed consent was obtained and the study was approved by the South East Wales Research Ethics committee (REC number: 10/WSE02/10). Placenta were collected within 2 h of an elective caesarean section (N = 32), an emergency caesarean section (N = 3) or a vaginal delivery (N = 7) from 42 term pregnancies (37–42 weeks). Chorionic villous samples were taken from the maternal side of the placenta at five different sites midway between the cord and lateral edge. To avoid contamination of the sample with maternal decidual cells, the top surface of the cotyledon was removed and the villous trophoblast tissue below sampled. Placental samples were washed in phosphate buffered saline and stored in RNAlater at −80 °C until needed. To analyse Intraplacental variation of telomere length, three placental samples (near to the cord insertion, middle and lateral edge) were biopsied from each of the fetal, middle and maternal layers as described by Wyatt et al. (2005) [29]. This was applied to five term placentas from elective C-section deliveries. Only placenta from healthy singleton Caucasian pregnancies with no recorded medical disorders and babies within the normal birth weight range were used in the study.

### 2.2 STELA (Single Telomere Length Analysis)

STELA was performed as described previously [25]. (Fig. 1). Briefly, Genomic DNA was isolated from approximately 40 mg of placental tissue by a standard proteinase K and phenol/chloroform protocol [30]. DNA was resuspended in 100 μl TRIS (10 mM, pH 8.0). A mean DNA yield of 70 μg was obtained per sample. DNA concentration was quantified in triplicate by Hoechst fluorometry and each DNA sample was then diluted to 10 ng/μl with 10 mM Tris (pH 8.0). Ten nanograms of DNA were diluted to 250 pg/μl in a final volume of 40 μl including 10 μM Telorette 2 primer and 10 mM Tris-HCl (pH 8.0) to generate the Tel2/DNA mix. Multiple reactions (usually six reactions per sample) were carried out for each test DNA in a final volume of 10 μl being composed of 250 pg of diluted DNA, Taq10xBuffer, MgCl2 (25 mM), NTPs (100 mM total), Teltail (5 μM) plus Telomere specific primer XpYpE2 (5 μM) (Table 1) and Taq (Life Technologies Ltd)/Pwo (Roche Molecular Biochemicals) (10:1). To avoid evaporation through the program cycles 10 μl of mineral oil was pipetted on the top of each reaction.

**Table 1:** STELA primer sequences.

Primer name	Oligonucleotide sequences
XpYpE2	5′-TTGTCTCAGGGTCCTAGTG-3′
Telorette2	5′-TGCTCCGTGCATCTGGCATCTAACCCT-3′
Teltail	5′-TGCTCCGTGCATCTGGCATC-3′

Thermal cycling conditions were: 22 cycles of 94 °C for 20 s, 65 °C for 30 s and 68 °C for 8 min. DNA was resolved on 0.5% 50 cm Tris-acetate-EDTA agarose gels alongside a 1 kb molecular weight marker (Agilent) and a 2.5 kb marker (Bio-Rad). DNA was transferred to a hybond-XL nylon membrane by Southern blotting and then hybridised with the TTAGGG repeat probe α-33P dCTP labelled (Perkin Elmer) and a probe to detect the DNA ladders. Hybridised fragments were detected by Typhoon FLA 9500 phosphoimager (GE Healthcare Life Sciences). Individual telomere lengths were measured using the ImageQuant software and descriptive statistics about the telomere length distributions were generated.

### 2.3 Statistics

All statistical analysis was performed using GraphPad Prism 7.02 (2016) for Windows. Non-normal distribution of the data was assessed using a nonparametric Mann-Whitney test. Student's t-test was also assessed showing non-evidence of significance.

## 3 Results

XpYp STELA was applied to a total of 42 unique placental samples to examine if the telomere length distributions varied according to intra-placental variation, mode of delivery and sex differences. A summary of participant demographics is given in Table 2.

**Table 2:** **Main characteristics of the study participants**. Mean (SD)/Range or number (%) is shown. Note: due to missing values, some numbers do not add up to 100%

Study Participants (N = 42)			
Maternal characteristic	Ethnicity (Caucasian)		39(93%)
	Parity	Primiparous	14(33.3%)
		Multiparous	24(57.1%)
	Maternal age		30(5.31)/20-40
	Maternal BMI		26(5.15)/17-38
Birth Outcome	Mode of Delivery	Vaginal	7(16.7%)
		Elective C section	32(76.2%)
		Emergency C section	3(7.1%)
	Birth weight (g)		3525(368)/3010-4580
	Gestational age (weeks)		39(1.15)/37-42
	Placental weight (g)		686(146)/396-1138
	Gender	Male	22(52.4%)
		Female	20(47.6%)

### 3.1 Intraplacental variation

**Fig. 2:**
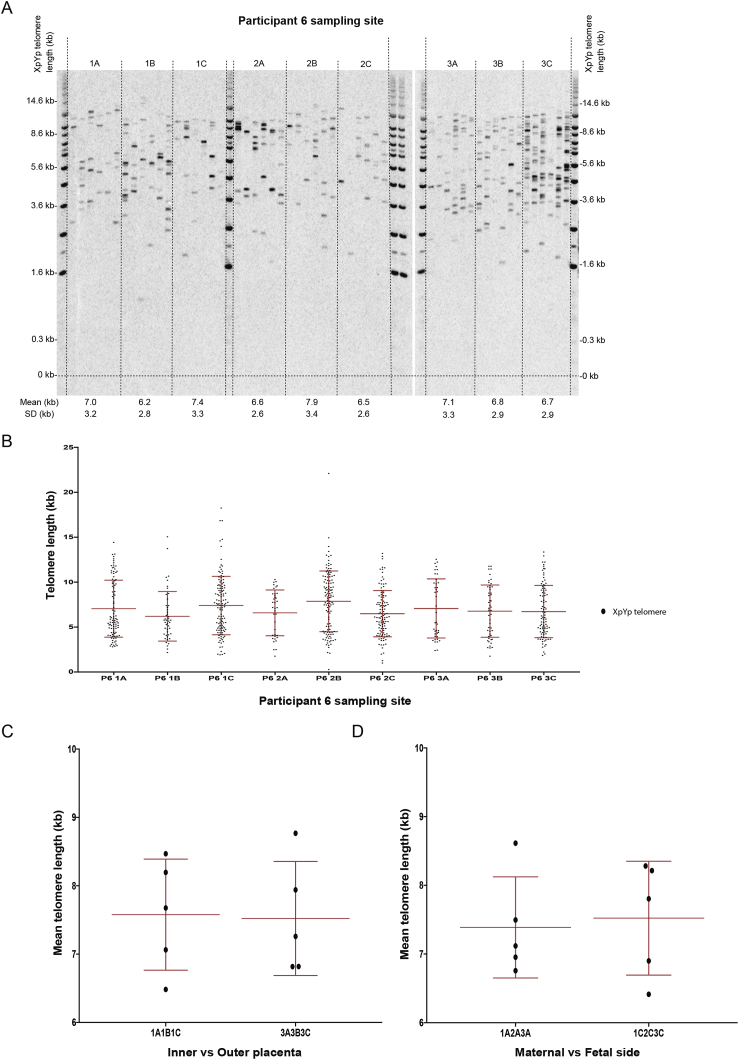
**Intra-placental variation in telomere length**. (A) STELA of nine genomic DNA samples from one placenta. (B) Graphical representation of data in A. (C) Comparison of mean telomere length between cord insertion (1A,1B,1C) and the lateral edge (3A,3B,3C). (D) Comparison of mean telomere length between the maternal (1A,2A,3A) and the fetal side (1C,2C,3C). Mean telomere length is presented (±SD). Five placenta were used for this study (N = 5). Mann–Whitney two-tailed test was used to assess statistical significant differences.*p < 0.05.

We previously showed that gene expression can vary with site of dissection [28]. Variation in telomere length with dissection site could introduce a significant confounder when comparing across different cohort studies. To examine intra-placental variation, STELA was performed on 9 different sampling sites (Supplementary Fig. 1) from N = 5 placentas. Results from one placental sample are shown in Fig. 2A with a graphical representation shown in Fig. 2B. There were no detectable differences in mean telomere length between cord insertion and lateral sampling sites (7.58 kb vs. 7.52 kb; p > 0.99) (Fig. 2C) or between the maternal and fetal side (7.39 kb vs. 7.52 kb; p > 0.99) (Fig. 2D).

### 3.2 Mode of delivery

**Fig. 3:**
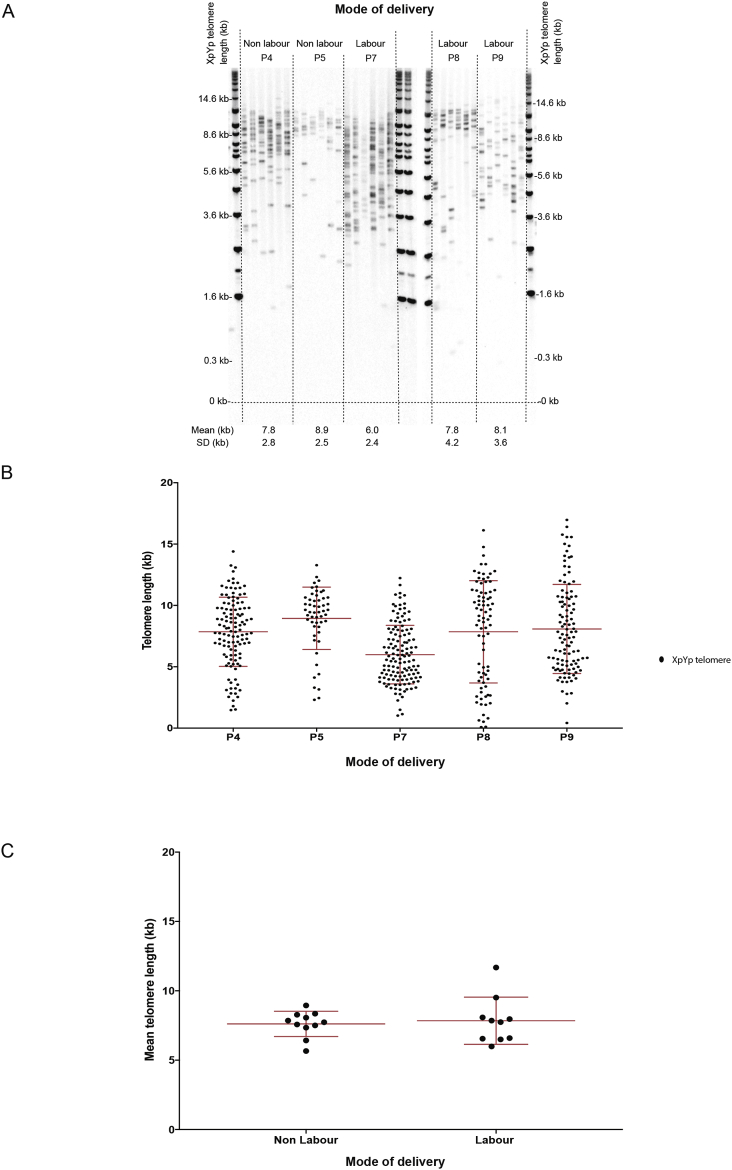
**Effect of labour on telomere length**. (A) STELA of five placental samples from five random participants. (B) Graphical representation of data in A. (C) Comparison of mean telomere length between non-labouring and labouring placental samples. Mean telomere length is presented (±SD). Twenty one participants were used for this study (N = 11 + 10). Mann–Whitney two-tailed test was used to assess statistical significant differences.*p < 0.05.

In addition to site of dissection, gene expression in the placenta can vary with mode of delivery [28]. Importantly, a recent study using qPCR in amniotic fluid samples reported that samples from term labour exhibited a higher telomere-repeat content than those not in labour at the point of sampling [31]. We applied STELA to labour (either vaginal delivery or emergency C-section; N = 10) and non-labour (elective C-section; N = 11) samples (Fig. 3A and B). There was no significant effect of labour on mean placental telomere length (7.85 kb vs. 7.61 kb; p = 0.97) (Fig. 3C).

### 3.3 Foetal sex

**Fig. 4:**
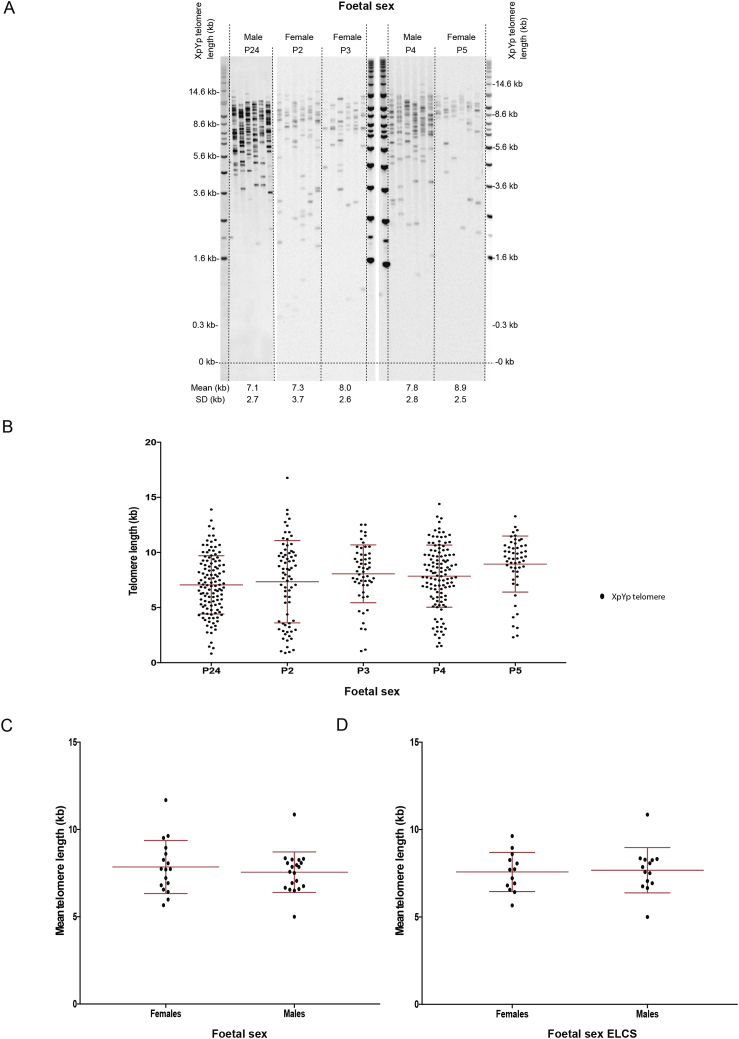
**Placental telomere length by foetal sex**. (A) Representative STELA of five placental samples and (B) their graphical representations. (C) Mean telomere length by foetal sex. (D) Mean telomere length for only the elective C-section placentas. Mean telomere length is presented (±SD). Thirty seven participants were used for this study (N = 17 + 20) and twenty seven when elective C-section samples analysed (N = 13 + 14). Mann–Whitney two-tailed test was used to assess statistical significant differences.*p < 0.05.

We then asked whether STELA identified subtle sex differences in placental telomere features by applying the technique to N = 17 female and N = 20 male placentas (Fig. 4A and B). The mean telomere length of female placenta was 300 bp longer than those observed in male placenta, but this difference was not significant (7.85 kb vs. 7.55 kb; p = 0.75) (Fig. 4C). There was also no significant difference in length when only the elective C-section samples were compared (N = 13 female and N = 14 male; 7.58 kb vs. 7.68 kb; p = 0.79) (Fig. 4D).

## 4 Discussion

Shorter telomeres have been correlated with some complications of pregnancy [15], [16], [17], [18], [19], [20], [21]. Some of the more commonly used techniques do not provide high-resolution analysis of telomere dynamics in single cells [24]. This is the first occasion on which STELA (Single Telomere Length Analysis) has been used to measure placental telomere length in a number of samples providing a much richer picture of telomere length homeostasis in this tissue.

In this study, we measured telomere length with respect to sampling site, mode of delivery and foetal sex. We measured the XpYp telomere located at the end of the ‘pseudoautosomal’ region that has an obligate crossover in each meiosis, and thus segregates independently of sex. We have assays for other unique telomeres such as 2p, 9q, 11q and 17p. However, previous work indicates that telomere distributions are similar between sex chromosomes and autosomal chromosomes thus we restricted this work to the XpYp telomere only [32]. Through STELA we were able to generate high-resolution telomere length profiles from placental samples. No significant difference was observed in telomere length in response to sampling site, mode of delivery or foetal sex.

Regarding placental sampling site, several studies have demonstrated intra-placental variation of gene expression by sampling site when assayed by qPCR [28]. As far as we are aware, only one study using TRF technique [33] has assessed mean telomere length to control to examine variability within the placenta. They reported no significant differences. Our study is consistent with their findings.

It has been reported that term labour amniotic fluid (AF) samples had a higher telomere-repeat content than term/not in labour AF [31] as assessed by qPCR, but the effect of mode of delivery on placental telomere length has not been reported. We did not find any correlation between mode of delivery and telomere length by STELA. We divided mode of delivery into two different categories: labour which includes vaginal delivery, emergency caesarean and forceps, and non-labour which refers solely to elective caesareans. Labour constitutes the natural end of a pregnancy whilst non-labour such as elective caesarean constitutes a planned procedure where labour itself has not yet begun. As a result, none of the signalling cascades which normally take place prior to the birth have initiated. It has been suggested that shortened telomeres trigger parturition [34] but our data does not support this theory, at least when examining the X and Y chromosomes. It is noteworthy to mention that the telomere length distributions observed across the samples showed that the cells within placenta contain telomeres within the length ranges observed in senescent cells [25]. These heterogeneous and short telomere length profiles are consistent with an extensive proliferative history of placental to generate this organ in a relatively short period of time.

We detected no significant differences in placental telomere length between males and females, either for the full set or just within the placenta from elective C-sections. This data is consistent with a previous study which reported no difference in placental telomere length between female and male within third trimester live-born twins [35]. Okuda et al. (2002) also reported no correlation between mean TRF and foetal sex when examining umbilical cord, foreskin or white blood cells [36]. However, a more recent study [37] reported a correlation between placental TL and sex, finding longer telomeres in term female placentas compared to male placentas by q-PCR. Adult women exhibit longer telomeres but this difference has not been universally reported in all studies [38].

The sample size in our study, although similar to the term placenta sample size in the Wilson study, may be too small to detect very subtle differences in telomere length. It is also possible that other methodological differences account for these findings. However, as we are examining only the X and Y chromosome telomeres, it may be that the sex chromosomes lack a sex difference in telomere length. Alternatively, there may be differences between populations. Further research is required.

In conclusion, this is the first study applying the STELA technique to measure placental telomere length distributions in relation to sampling site, mode of delivery and foetal sex. In this study, we found no significant differences by these criteria, which indicate that this technique can be used widely in pregnancy cohort studies irrespective of study design. The small sample size is a limitation of this study. Further statistical analysis and additional modelling with respect to other variables, including gestational age, in a much larger sample set is required to confirm these findings. Nonetheless, we noted a very high level of heterogeneity within samples not previously been reported for the placenta. Given that this is a transient organ of pregnancy that lasts a mere 9 months, it is remarkable that such heterogeneity is generated. This technique reveals a much richer picture of telomere dynamics which will be important for future studies exploring the relationship between telomere length with birth outcomes and maternal lifestyles.

## Conflict of interest statement

On behalf of all the authors, the corresponding author, Rosalind M John, declares there are no conflicts of interest related to the research paper “**Telomere length heterogeneity in placenta revealed with high-resolution telomere length analysis**”

## Appendix A Supplementary data

Supplementary material **Sampling site dissection**. Adapted from Wyatt et al. Placenta (2005). From the medial to the lateral edge the tissue was divided into three fractions and each of these fractions was in turn divided into three sections from the chorionic plate to the basal plate, obtaining 9 sampling sites per placenta for a total of 5 placentas. Supplementary material
