# Supplementary figures and images for: Telomere length heterogeneity in placenta revealed with high-resolution telomere length analysis
Source: Placenta. 2017 Nov;59:61–8. doi: 10.1016/j.placenta.2017.09.007 (PMC5687939; doi:10.1016/j.placenta.2017.09.007)

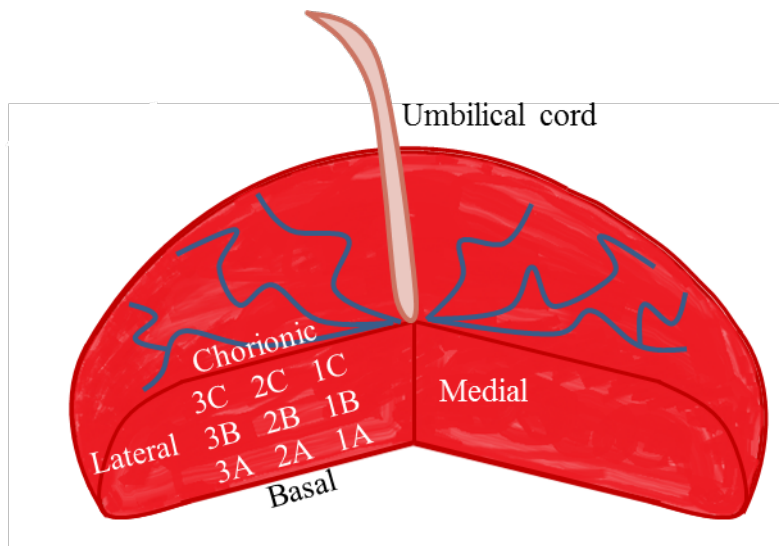

Supplement: Supplementary material — Sampling site dissection. Adapted from Wyatt et al. Placenta (2005). From the medial to the lateral edge the tissue was divided into three fractions and each of these fractions was in turn divided into three sections from the chorionic plate to the basal plate, obtaining 9 sampling sites per placenta for a total of 5 placentas. [file mmc1.pdf]
